# Publisher Correction: Specific 3-*O*-sulfated heparan sulfate domains regulate salivary gland basement membrane metabolism and epithelial differentiation

**DOI:** 10.1038/s41467-024-53230-4

**Published:** 2024-10-10

**Authors:** Vaishali N. Patel, Marit H. Aure, Sophie H. Choi, James R. Ball, Ethan D. Lane, Zhangjie Wang, Yongmei Xu, Changyu Zheng, Xibao Liu, Daniel Martin, Jillian Y. Pailin, Michaela Prochazkova, Ashok B. Kulkarni, Toin H. van Kuppevelt, Indu S. Ambudkar, Jian Liu, Matthew P. Hoffman

**Affiliations:** 1 Matrix and Morphogenesis Section, National Institute of Dental and Craniofacial Research, NIH, DHHS, Bethesda, MD USA; 2 Division of Chemical Biology and Medicinal Chemistry, Eshelman School of Pharmacy, University of North Carolina, Chapel Hill, NC USA; 3 Glycan Therapeutics Corp, Raleigh, NC USA; 4 Translational Research Core, National Institute of Dental and Craniofacial Research, NIH, DHHS, Bethesda, MD USA; 5 Secretory Physiology Section, National Institute of Dental and Craniofacial Research, NIH, DHHS, Bethesda, MD USA; 6 NIDCD/NIDCR Genomics and Computational Biology Core, National Institute of Dental and Craniofacial Research, NIH, DHHS, Bethesda, MD USA; 7 Functional Genomics Section, National Institute of Dental and Craniofacial Research, NIH, DHHS, Bethesda, MD USA; 8 Department of Biochemistry, Radboud University Medical Center, Nijmegen, The Netherlands

**Keywords:** Differentiation, Stem-cell niche, Glycobiology

Correction to: *Nature Communications* 10.1038/s41467-024-51862-0, published online 31 August 2024

The original version of this Article contained an error in Fig. 2c, in which the bar chart for the “Male DKO” condition was white in colour while it should have been grey to correspond with the legend. Likewise, the original version of this Article contained errors in Fig. 4c and 4d, in which the bar charts for the “WT” condition were coloured white while they should have been in grey to correspond with the legends. These have been corrected in both the PDF and HTML versions of the Article.

