# Supplementary material for: Specific 3-O-sulfated heparan sulfate domains regulate salivary gland basement membrane metabolism and epithelial differentiation
Source: Nat Commun. 2024 Aug 31;15:7584. doi: 10.1038/s41467-024-51862-0 (PMC11365954; doi:10.1038/s41467-024-51862-0)
Supplement: Supplementary file 1 — Supplementary Information [file 41467_2024_51862_MOESM1_ESM.pdf]

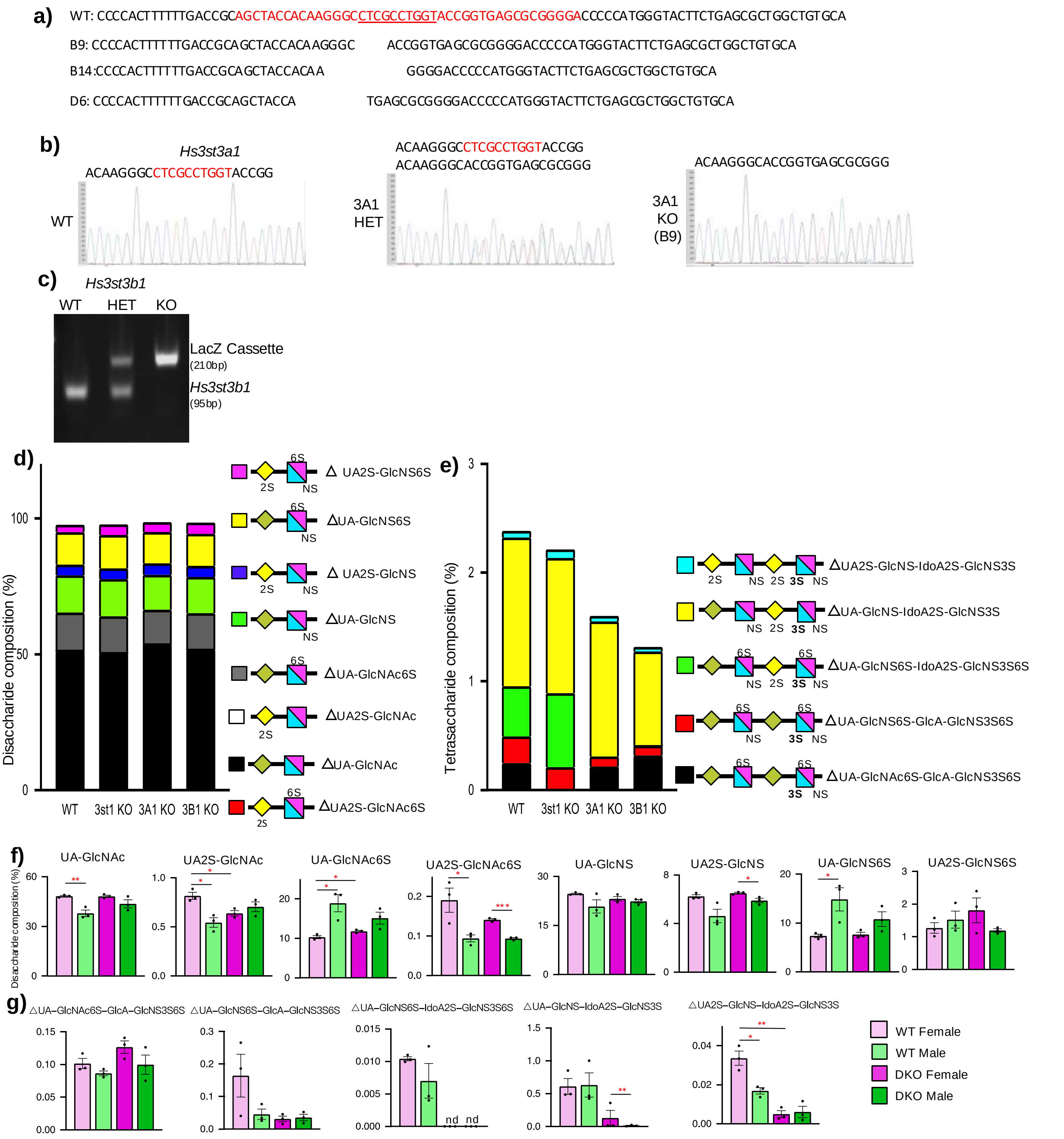

Supplementary Fig. 1. Generation of *Hs3st3a1*;*Hs3st3b1* DKO mice

a Sequence alignment of ZFN-induced deletions in *Hs3st3a1* sequence from three different founders compared to the WT sequence. Deletion length: B9=10bp, B14=26bp, D6=22bp. The red letters are the ZFN binding site and the underlined letters are the ZFN cut site. **b** Direct sequencing of the *Hs3st3a1* PCR amplicons from the founder line D6. **c** Genotyping for detection of *Hs3st3b1* PCR product based on detection of the WT allele (lower band) and the targeting vector (Neomycin cassette- upper band). **d-f** Mass spec analysis of HS disaccharides and tetrasaccharides in adult SMGs. Analysis of HS disaccharide (**d**) and tetrasaccharide (**e**) composition between WT and *Hs3st1*, *Hs3st3a1* (3A1) and *Hs3st3b1* (3B1) single KO male SMGs. △UA2S-GlcNAc6S not detected in SMGs. △UA-GlcNS6S-IdoA2S-GlcNS3S6S not detected in *Hs3st3a1* (3A1) and *Hs3st3b1* (3B1) SMGs. △UA-GlcNAc6S-GlcA-GlcNS3S6S not detected in *Hs3st1* KO. 3-4 glands were pooled together for each sample, *n*=3 samples per group and stacked graphs are showing average for each group. Paired two-tailed t-test performed. **f-g** Analysis of HS disaccharides (**f**) and tetrasaccharides (**g**) of adult female and male WT and DKO SMGs. For the HS analysis, 3-4 glands were pooled together for each sample, *n*=3 samples per group and graphs are showing average for each group. Unpaired two-tailed t-test performed. WT female vs WT male: UA-GlcNAc; $**p=0.0089$, UA2S-GlcNAc; $*p=0.0156$, UA-GlcNAc6S; $*p=0.0101$, UA2S-GlcNAc6S; $*p=0.0261$, UA-GlcNS6S; $*p=0.0216$ and UA2S-GlcNS-IdoA2S-GlcNS3S; $*p=0.0102$. DKO female vs DKO male: △UA2S-GlcNAc6S; $***p=0.0002$ and △UA2S-GlcNS; $*p=0.0378$. nd = not detected. Source data are provided as Source Data file.

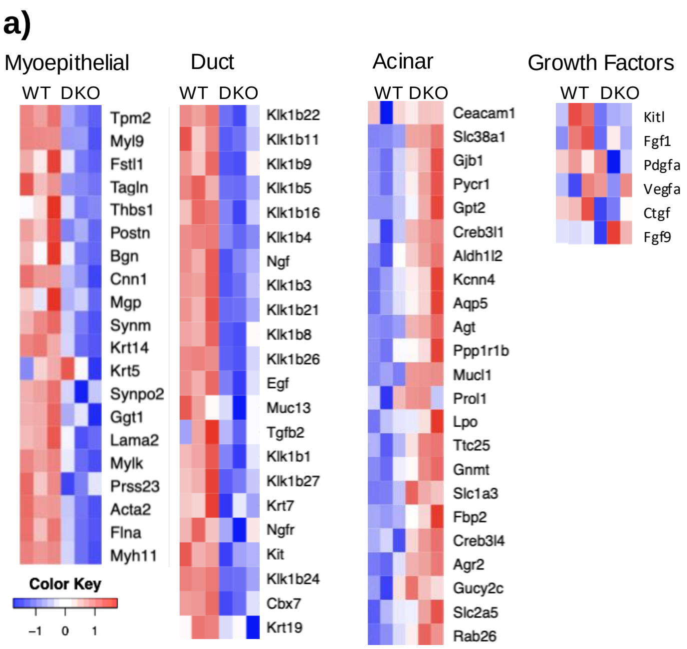

Supplementary Fig. 2. RNAseq analysis of female DKO SMGs also showed reduced MEC and duct gene expression and increased expression of acinar gene, while GO analysis suggests cell processes that may be affected.
a Heat maps of DEGs expressed by MEC, ductal and acinar cells in female DKO SMGs (*n*=3) compared to WT (*n*=3). The color scale represents scaled gene expression values. DEGs met the criteria for significance in our dataset (*p*-Value < 0.05 and fold change >2) obtained using a non-parametric Wald test with Benjamini-Hochberg adjustment.

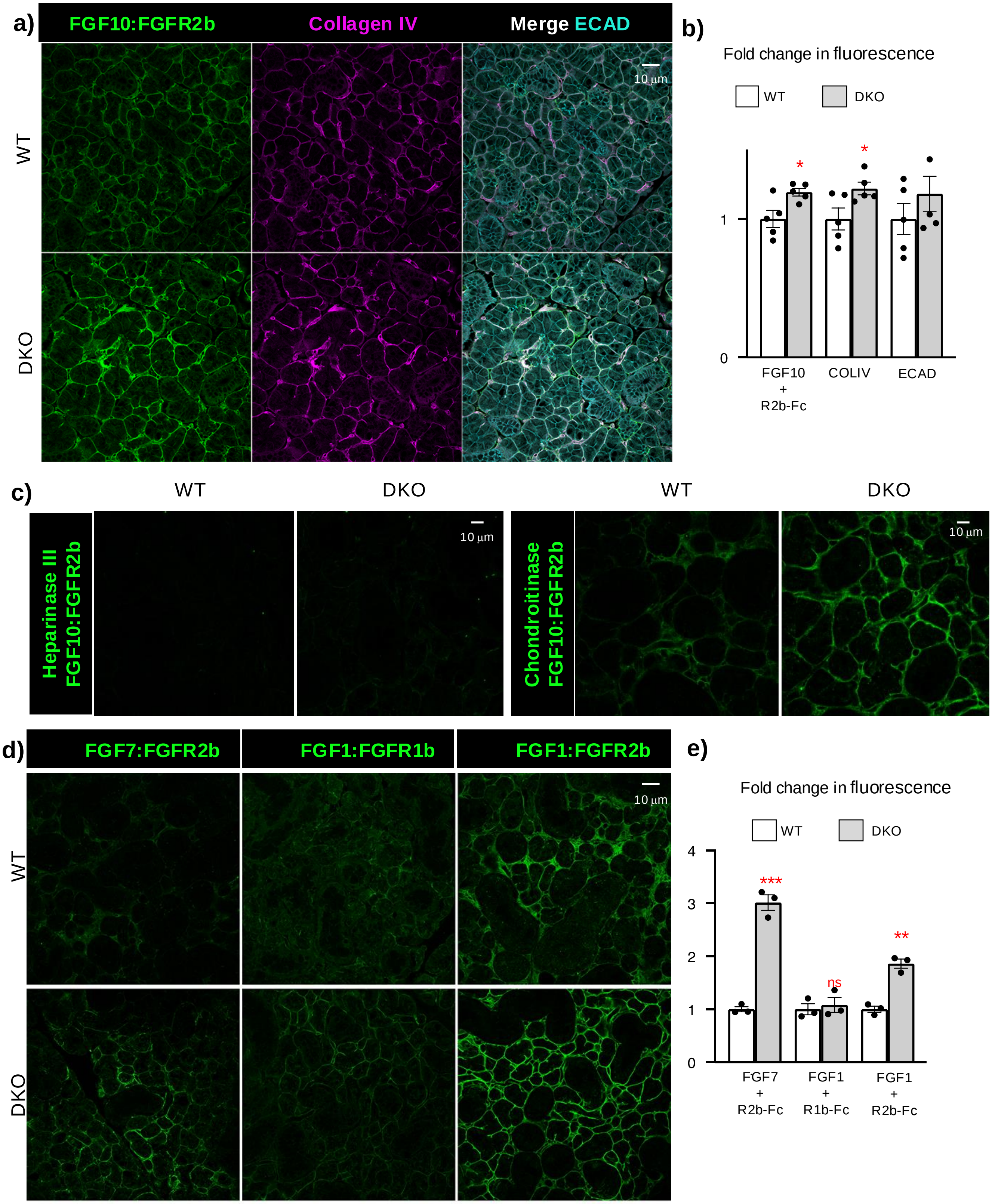

Supplementary Fig. 3. LACE assay binding is HS-dependent and other FGF:FGFR complexes also increase binding to DKO HS.
a Binding of FGF10:FGFR2b-Fc complex overlapping with COLIV, a basement membrane protein, is increased in DKO female SMGs. Representative images of single confocal sections from WT and DKO SMGs showing FGFR2b-Fc (green), COLIV (magenta), and E-cadherin (ECAD, cyan). Scale bar: 10 μm. **b** Quantification of A and B protein fluorescence intensity normalized to total nuclei and expressed as a fold change compared to WT. *n*=5 SMGs for each genotype. Error bars: SM. Unpaired two-tailed t-test compared to WT, LACE ***p*=0.0229, COLIV **p* = 0.0450. **c** Heparinase III and chondroitinase ABC treatment confirms the binding of FGF10:FGFR2b-Fc is dependent on HS. Representative images of single confocal sections from WT and DKO SMGs showing FGF1:FGFR2b-Fc complex (green) binding on sections pretreated with heparinase III is abolished but not with chondroitinase ABC treatment. *n*=3 SMGs. Scale bar: 10 μm. **d** Binding of FGF1:FGFR2b-Fc and FGF7:FGFR2b-Fc complexes, but not FGF1:FGFR1b-Fc, to basement membrane HS is increased in *Hs3st3* DKO male SMGs. Representative images of single confocal sections from WT and *Hs3st3* DKO SMGs showing FGFR2b-Fc (green). Scale bar: 10 μm. **e** Quantification of protein fluorescence intensity normalized to total nuclei and expressed as a fold change compared to WT. SMGs from three male mice for each genotype were imaged and used for quantification. Error bars: SM. *n*=3 SMGs for each genotype. Unpaired two-tailed t-test compared to WT, ****p* < 0.0001 for FGF7:FGFR2b-Fc, ** *p* < 0.0012 or not significant (ns). Source data are provided as Source Data file.

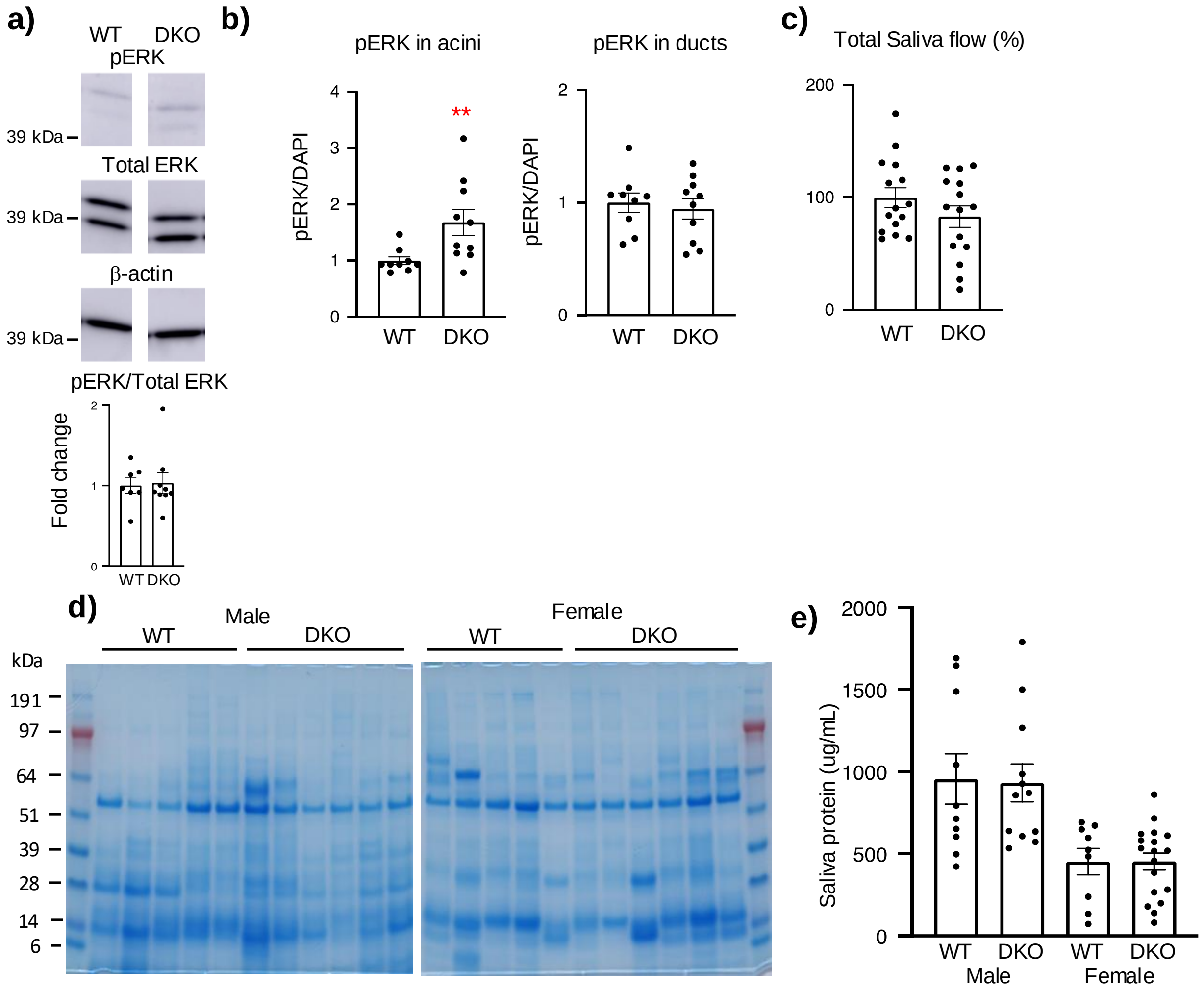

Supplementary Fig. 4. Analysis of saliva proteins from WT and DKO mice.
a Western blot showing phospho-Erk in total lysate of female DKO SMGs compared to WT. Representative blot of *n*=7 WT and *n*=9 DKO SMG lysates. Source data with full blots are provided as Source Data file. **b** Quantification of pERK staining in acini and ducts normalized to nuclei shown as fold change normalized to WT. Graph shows Mean ± SM. *n*=9 (WT) and *n*=10 (DKO) SMGs. Unpaired two-tailed t-test compared to WT, ** p= 0.0092. **c** Salivary flow rates in adult female mice collected after pilocarpine stimulation. Saliva flow normalized to the WT and shown as %. Mean ± SM. *n*=15 WT and *n*=15 DKO mice. Unpaired two-tailed t-test, ns. **d** There are no obvious differences in protein expression profiles in DKO SMGs. Saliva (15 µl) from female and male mice were analyzed by 4-12% gradient SDS-polyacrylamide gels stained with Coomassie Blue. *n*=5 (WT-male), *n*=6 (DKO-male), *n*=5 (WT-female), *n*=6 (DKO-female) mice saliva samples. Each lane represents individual biological sample. **e** Quantification of protein concentration in saliva assessed using BCA assay. Graph shows Mean ± SM. *n*=10 (WT-male), *n*=12 (DKO-male), *n*=9 (WT-female), *n*=18 (DKO-female) mice saliva samples. No significant differences detected compared to WT samples using unpaired two-tailed t-test. Source data are provided as Source Data file.

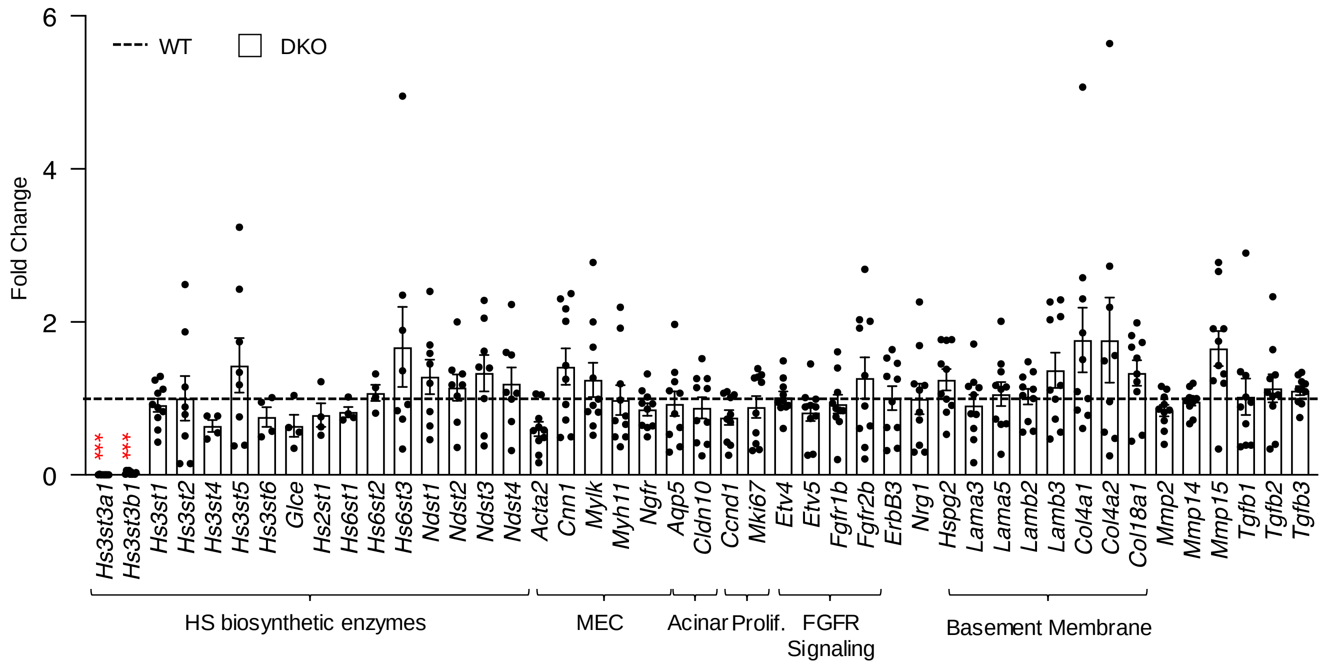

Supplementary Fig. 5. qPCR of E13 DKO SMGs does not reveal major transcriptional changes.
Gene expression changes in freshly dissected E13 DKO SMGs were normalized to WT control and *Rps29*. Error bars: SM. *n*=9 SMGs for WT except for *Hs3st3a1, Ndst1, Ndst2, Ndst3, Ndst4: n*=8, *Hs3st5: n*=7 and *Hs3st4*, *Hs3st6*, *Glce*, *Hs2st1*, *Hs6st1* and *Hs6st2*: *n*=4. *n*=10 SMGs for DKO except for *Hs3st3a1, Hs3st2, Hs6st3, Ndst1, Ndst2, Ndst3, Ndst4: n*=8, and *Hs3st4, Hs3st6, Glce*, *Hs2st1*, *Hs6st1* and *Hs6st2*: *n*=4. Unpaired two-tailed t-test compared to WT, *Hs3st3a1* $^{***}p < 0.0001$, *Hs3st3b1* $^{***}p <$ 0.0001. Source data are provided as Source Data file.

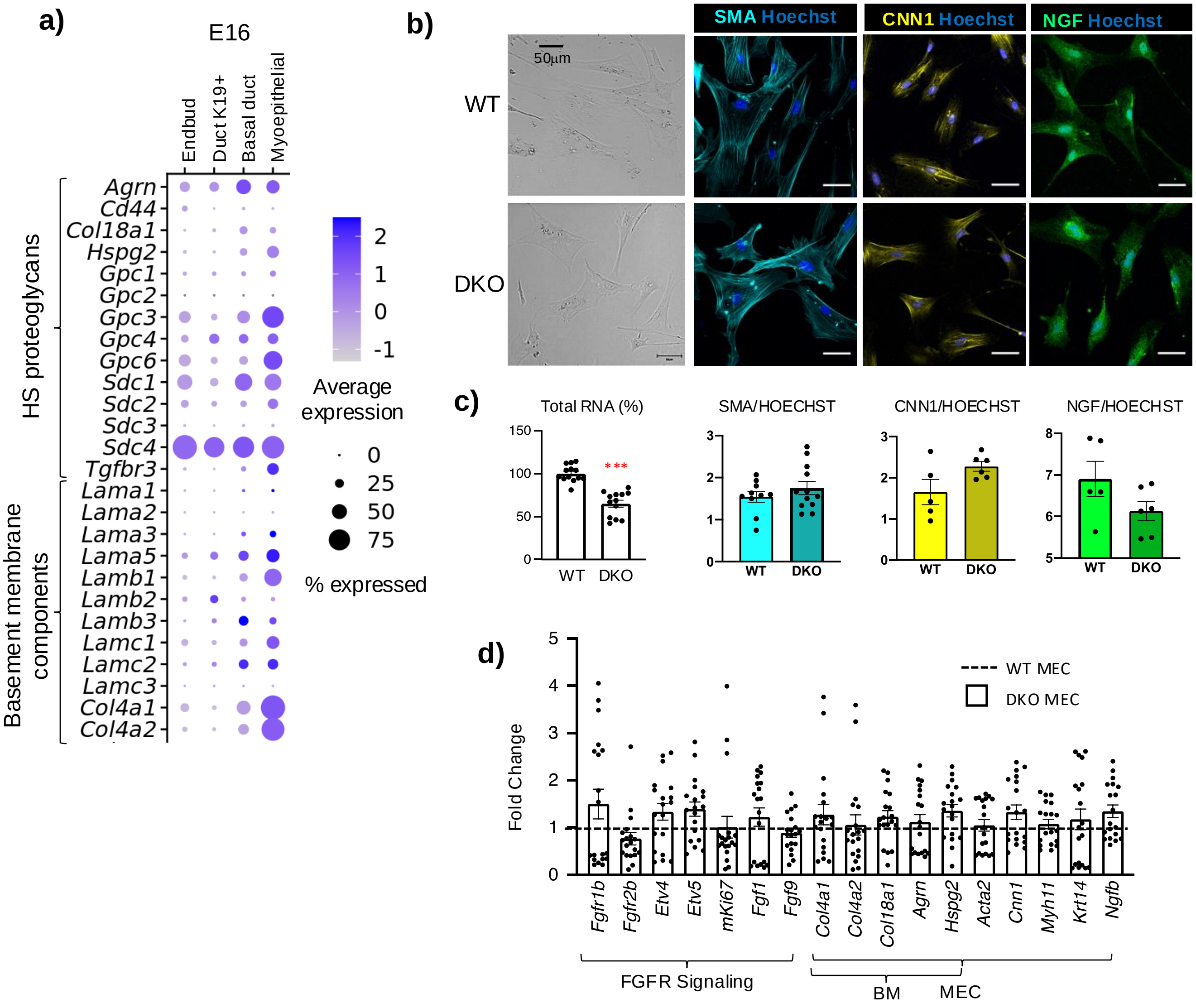

Supplementary Fig. 6. P2 DKO MECs cultured on collagen IV for 7 days are similar to WT MECs in gene expression and appearance, but the RNA concentration is decreased.

a Dot plot of HSPGs and basement membrane expression from E16 SMG scRNA seq data (GSE150327). **b** Brightfield image of WT and DKO MEC cultured on collagen IV for 7 days. Scale bar: 50 µm. Representative images of WT and DKO MEC cultured on collagen IV for 7 days stained with SMA (cyan), CNN1 (yellow) or NGF (green). Scale bar = 50 µm. *n*=3 independent experiments. **c** Quantification of RNA is shown in %. Data for each experiment normalized to WT. WT *n*=13 and DKO *n*=13. Error bars: SM. Unpaired two-tailed t-test compared to WT, ****p* < 0.0001. Quantification of immunostaining showed similar protein expression levels in DKO compared to WT. Each datapoint is one area. Error bars: SM. **d** Gene expression of isolated P2 MEC cultured for 7 days on collagen IV in MEC growth media. Data for each experiment normalized to WT. WT *n*=6 with 16 total replicate wells and DKO *n*=5 with 19 total replicate wells. Error bars: SM. Unpaired two-tailed t-test compared to WT, ns. Source data are provided as Source Data file.

a)

Weaned mice from *Hs3st1* X *Hs3st3b1*

Hs3st3b1 \ *Hs3st1*	WT	HET	KO
WT	9	14	11
HET	20	46	11
KO	8	17	0

E13 embryos from *Hs3st1* X *Hs3st3b1*

Hs3st1 \ *Hs3st3b1*	WT	HET	KO
WT	6	12	3
HET	6	23	13
KO	6	3	1

E11.5 embryos from double HET *Hs3st1* X *Hs3st3b1*

Hs3st3b1 \ *Hs3st1*	WT	HET	KO
WT	1	1	0
HET	1	5	2
KO	2	1	0

E11.5 embryos from *Hs3st1*$^{-/-}$;*Hs3stsb1*$^{+/-}$ X DHET

Hs3st3b1 \ *Hs3st1*	WT	HET	KO
WT	0	0	0
HET	4	4	1
KO	5	5	3

b) *Hs3st1*;*Hs3st3b1* WT *Hs3st1*;*Hs3st3b1* DHET *Hs3st1*;*Hs3st3b1* DKO

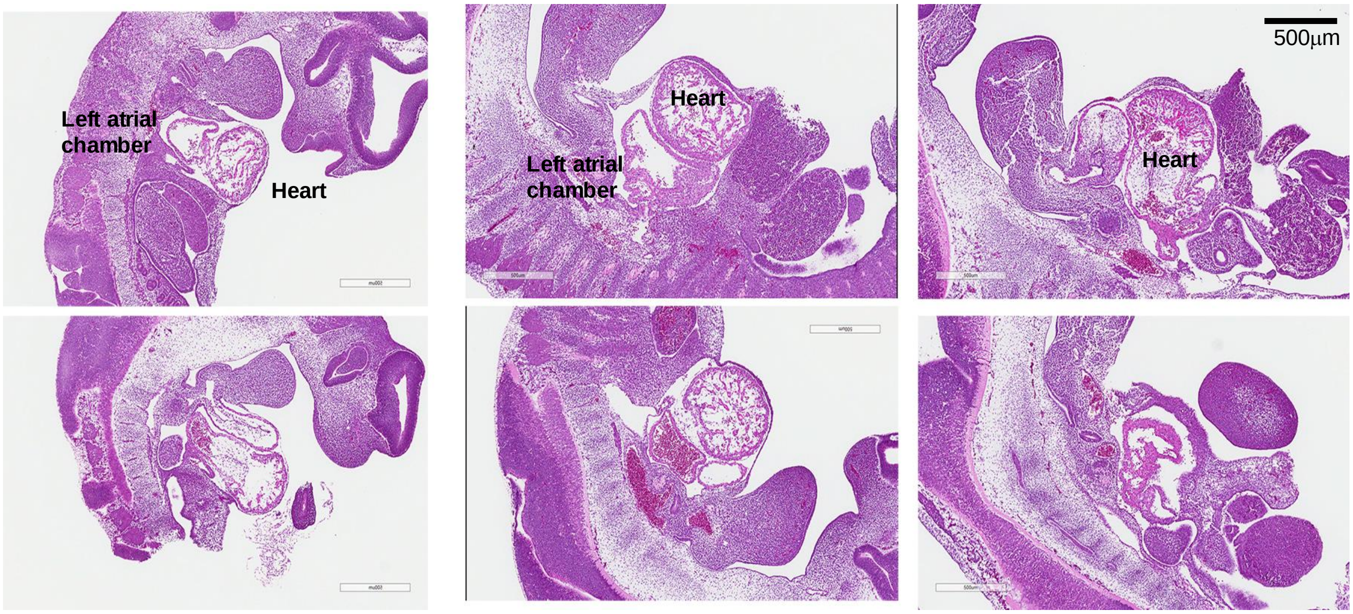

Supplementary Fig.7. *Hs3st1*; *Hs3st3b1* DKO mice die at ~E11.5

a Analysis of breeding crosses to obtain *Hs3st1;Hs3st3b1* DKO embryos. **b** *Hs3st1;Hs3st3b1* DKO embryos develop until ~11.5. Two sagittal sections of each genotype stained with H&E suggest that heart development is disrupted, which may lead to embryonic lethality. Representative images of *n*=3 embryos. Scale bar: 500 µm.

Supplementary Table 1a. KEGG pathways in male *Hs3st3a1; Hs3st3b1* DKO SMGs (related to Fig. 2).

Term	P-value	# Genes	Genes
Renin-angiotensin system	4.57E-10	12	KLK1B11;KLK1B8;KLK1B22;KLK1B21;KLK1B9;KLK1;KLK1B26;KLK1B3;KLK1B24;KLK1B5;KLK1B1;AGT
Endocrine and calcium reabsorption	9.35E-08	12	KLK1B8;KLK1B11;KLK1B22;KLK1B21;KLK1B9;KLK1;KLK1B26;KLK1B3;KLK1B24;FXYD2;KLK1B5;KLK1B1
**PI3K-Akt signaling pathway**	8.73E-07	30	LAMA2;ITGB3;LPAR1;TNC;PDGFA;TGFA;FGF1;THBS1;HSP90B1;KLK1B4;FGF9;CREB3L4;CREB3L1;PDGFD;GNG7;AKT3;CHAD;ITGB6;EIF4B;PCK2;YWHAH;NGFR;LAMB3;EGF;NGF;PRLR;VEGFA;NR4A1;COL6A2;SGK1
**Focal adhesion**	1.09E-06	21	JUN;SHC2;PPP1R12A;LAMA2;LAMB3;EGF;ITGB3;TNC;PDGFA;THBS1;MYL12A;MYLK;VEGFA;PAK1;PDGFD;COL6A2;AKT3;CHAD;FLNA;ITGB6;MYL9
**MAPK signaling pathway**	5.04E-05	23	DUSP4;NGFR;JUN;TGFB2;GADD45A;EGF;PDGFA;TGFA;FGF1;NGF;VEGFA;MAPK13;NR4A1;PAK1;CACNB3;KLK1B4;FGF9;PDGFD;AKT3;RPS6KA1;FLNA;CD14;MAPT
Thyroid hormone synthesis	8.27E-05	10	TTR;HSPA5;CREB3L4;CREB3L1;FXYD2;DUOXA2;GPX7;DUOX2;PDIA4;HSP90B1
Synaptic vesicle cycle	1.31E-04	11	UNC13B;ATP6V1G1;SLC6A9;STXBP1;SLC1A2;SLC1A3;STX3;ATP6V1E1;ATP6V0E;ATP6V1C2
Collecting duct acid secretion	1.48E-04	6	ATP4A;ATP6V1G1;ATP6V1E1;SLC12A7;ATP6V1C2;ATP6V0E
**ECM-receptor interaction**	2.45E-04	10	SV2C;LAMA2;LAMB3;ITGB3;COL6A2;CHAD;TNC;ITGB6;THBS1;CD44
Protein digestion and absorption	4.75E-04	10	KCNK5;DPP4;SLC15A1;SLC7A7;COL6A2;FXYD2;SLC3A2;SLC1A5;KCNN4;SLC38A2
Glutathione metabolism	7.90E-04	8	GSTM2;GCLC;NAT8;GSTO1;NAT8F1;G6PDX;GPX7;GGT1
**Regulation of actin cytoskeleton**	0.0013	16	PPP1R12A;EGF;ITGB3;LPAR1;PDGFA;FGF1;MYL12A;MYLK;PAK1;FGF9;PDGFD;TMSB4X;ITGB6;EZR;MYL9;PFN2
Glycine, serine and threonine metabolism	0.0014	6	SHMT2;PSAT1;SARDH;PHGDH;PSPH;GNMT
Phagosome	0.0016	14	ATP6V1G1;ITGB3;STX7;THBS1;DYNC1LI2;TUBB2B;TUBA1A;TUBB2A;CTSL;CD14;NOS1;ATP6V1E1;ATP6V1C2;ATP6V0E
**Proteoglycans in cancer**	0.0017	15	TGFB2;CAMK2D;PPP1R12A;ITGB3;THBS1;VEGFA;MAPK13;PAK1;CTSL;AKT3;FLNA;EZR;CD44;EIF4B;WNT4
**Adrenergic signaling**	0.0022	12	CACNB3;CAMK2D;CREB3L4;CREB3L1;TPM2;FXYD2;AKT3;TPM1;ADRA1A;SCN1B;AGT;MAPK13

Supplementary Table 1b. KEGG pathways in female *Hs3st3a1; Hs3st3b1* DKO SMGs (Related to Fig. 2).

Term	P-value	# Genes	Genes
Renin-angiotensin system	1.35E-12	13	KLK1B11;KLK1B22;KLK1B21;MME;KLK1B26;KLK1B24;LNPEP;AGT;KLK1B8;KLK1B9;KLK1B3;KLK1B5;KLK1B1
Hematopoietic cell lineage	1.20E-11	18	CSF3R;ITGA4;MME;ITGB3;CD3G;IL6RA;H2-AA;H2-EA-PS;H2-DMB2;CD4;CD8A;KITL;KIT;H2-OB;CD37;IL7R;CD22; H2-AB1
**Cell adhesion molecules (CAMs)**	3.99E-08	19	CD274;VCAM1;ITGA4;SDC2;SDC3;H2-Q4;ITGAL;H2-AA;ICAM1;H2-EA-PS;H2-DMB2;CD4;CD8A;H2-OB;ITGA8;SIGLEC1;H2-D1;CD22;H2-AB1
Staphylococcus aureus infection	7.77E-08	14	C1QB;C1QA;CFH;FGG;ITGAL;H2-AA;ICAM1;C4B;H2-EA-PS;H2-DMB2;C1S1;FCGR4;H2-OB;H2-AB1
Malaria	2.48E-07	10	VCAM1;LRP1;SDC2;HBA-A2;HBB-BT;HBA-A1;HBB-BS;ITGAL;THBS2;ICAM1
Aminoacyl-tRNA biosynthesis	5.43E-07	11	CARS;NARS;YARS;VARS;RARS;MARS;SARS;TARS;GARS;EPRS;AARS
Endocrine and calcium reabsorption	7.74E-07	10	KLK1B8;KLK1B11;KLK1B22;KLK1B21;KLK1B9;KLK1B26;KLK1B3;KLK1B24;KLK1B5;KLK1B1
Th1 and Th2 cell differentiation	8.92E-06	12	H2-EA-PS;H2-DMB2;CD4;MAF;NOTCH1;LCK;IL2RB;H2-OB;CD3G;H2-AA;H2-AB1
Viral myocarditis	8.92E-06	11	H2-EA-PS;H2-DMB2;LAMA2;H2-OB;H2-Q4;FYN;ITGAL;H2-AA;H2-D1;ICAM1;H2-AB1
Phagosome	9.66E-06	16	COLEC12;ITGB3;H2-Q4;CYBB;THBS2;H2-AA;H2-EA-PS;SEC61A1;H2-DMB2;FCGR4;MRC1;H2-OB;ATP6V0A4;TLR6;H2-D1;H2-AB1
Antigen processing and presentation	1.24E-05	11	H2-EA-PS;H2-DMB2;CD74;CIITA;CD4;CD8A;H2-OB;H2-Q4;H2-AA;H2-D1;H2-AB1
**Rap1 signaling pathway**	1.66E-05	17	PDGFRA;EGF;ITGB3;PIK3CD;CALML3;PIK3R1;ITGAL;NGF;ADCY7;RAP1GAP;KLK1B4;KITL;KIT;P2RY1;LCP2;SKAP1;EPHA2
Natural killer cell cytotoxicity	3.32E-05	12	VAV3;TYROBP;KLRK1;FCGR4;LCK;PIK3CD;LCP2;FYN;PIK3R1;ITGAL;H2-D1;ICAM1
**Focal adhesion**	3.37E-05	16	VAV3;PDGFRA;PPP1R12A;ITGA4;LAMA2;EGF;ITGB3;PIK3CD;PIK3R1;THBS2;MYLK;ITGA8;FLNA;FYN;MYL9;VCL
Human T-cell leukemia virus 1 infection	3.70E-05	18	H2-Q4;PIK3CD;CD3G;PIK3R1;ITGAL;ADCY7;H2-AA;ICAM1;H2-EA-PS;H2-DMB2;CD4;CREB3L4;LCK;CREB3L1;IL2RB;H2-OB;H2-D1;H2-AB1
Systemic lupus erythematosus	5.17E-05	13	C1QB;C1QA;H2-AA;C4B;H2-EA-PS;H2-DMB2;C1S1;FCGR4;H2AFJ;H2-OB;TRIM21;HIST1H2BC;H2-AB1
Leukocyte migration	1.23E-04	11	VAV3;ITK;VCAM1;ITGA4;CYBB;PIK3CD;PIK3R1;ITGAL;MYL9;VCL;ICAM1
T cell receptor signaling pathway	1.86E-04	10	VAV3;ITK;CD4;CD8A;LCK;PIK3CD;CD3G;LCP2;FYN;PIK3R1
Th17 cell differentiation	2.02E-04	10	H2-EA-PS;H2-DMB2;CD4;LCK;IL2RB;H2-OB;CD3G;IL6RA;H2-AA;H2-AB1
**PI3K-Akt signaling pathway**	2.12E-04	21	PDGFRA;CSF3R;ITGA4;LAMA2;EGF;ITGB3;PIK3CD;IL6RA;PIK3R1;NGF;THBS2;KLK1B4;CREB3L4;KITL;CREB3L1;KIT;IL2RB;EIF4EBP1;ITGA8;IL7R;EPHA2
Rheumatoid arthritis	2.13E-04	9	H2-EA-PS;H2-DMB2;H2-OB;ATP6V0A4;LTB;ITGAL;H2-AA;ICAM1;H2-AB1

The *p*-values for KEGG analysis tables are derived using right-tailed Fisher's exact t-test followed by a Benjamini-Hochberg adjustment.

Supplementary Table 2

**REAGENT/RESOURCE**	**SOURCE**	**IDENTIFIER**	**Dilution**
**Antibodies**			
HS4C3V single-chain HS Ab	Toin H. van Kuppevelt (Radboud University Medical Center, The Netherlands)	N/A	1:50
Anti-VSV glycoprotein-Cy3	Sigma	C7706	1:100
Antithrombin III labeled with Alexa Fluor 488 (AT488)	Dr. Nicholas Shworak (George Washington University)	N/A	100 nM
Anti-heparan sulfate proteoglycan (perlecan)	Millipore Sigma	mAb1948	1:200
Anti-E-cadherin (2AE10)	Cell Signaling	mAb#3195	1:100
Anti-E-cadherin	BD Biosciences	#610182	1:200
Anti-Smooth muscle actin	Millipore Sigma	A2547	1:200
Anti-Aquaporin 5	Alomone Labs	AQP-005	1:100
Acinar-1 antibody	DSHB	3.7A12	1:100
Anti-calponin (CNN1)	Abcam	ab46794	1:100
Anti-keratin 14	BioLegend	PRB-155P	1:2000
Anti-Mucin 13	Santa Cruz Biotechnology	Sc-390115	1:100
Anti-Mucin10 (Prol1)	Everest Biotech	EB10617	1:200
Anti-Collagen TIV	Millipore Sigma	AB769 (goat)	1:200
Anti-Collagen TIV	Millipore Sigma	AB756 (rabbit)	1:200
Anti-NGF	Alomone Labs	AN-240	1:100
Anti-agrin	R & D	AF550	1:100
Anti-beta-actin	Santa Cruz Biotechnology	Sc-47778	1:5,000
p44/p42 (Erk1/2)	Cell signaling	#9102	1:2000
phospho-p44/p42 MAPK (Erk1/2) (Thr202/Tyr204)	Cell signaling	#4370	1:1000 (WB) 1:100 (IF)
tight junction protein 1 ZO-1	Invitrogen	339100	1:200
anti-Delta-heparan sulfate 3G10	Asmbio LLC	370260-1	1:2000 (WB) 1:200 (IF)
Anti-EGF	Dr. Edward W.Gresik		1:100
All dye-conjugated secondary antibodies	Jackson ImmunoResearch Laboratories		1:200
Alexa Fluor® 488 AffiniPure $F(ab')_2$ Fragment Donkey Anti-Human IgG, Fcγ fragment specific antibody	Jackson ImmunoResearch Laboratories	709-546-098	1:200
Anti-mouse IgG, HRP-linked antibody	Cell Signaling	#7076	1:10,000
Anti-rabbit IgG, HRP-linked antibody	Cell Signaling	#7074s	1:10,000
Beta-actin (13E5) Rabbit mAb (HRP conjugate)	Cell Signaling	#5125S	1:1,000
**Chemicals, recombinant proteins, and enzymes**			
DAPI (Dihydrochloride)	Millipore Sigma	268298	1:10000
Hoechst	Jackson Immunoresearch Laboratories	715-165-150	1:10,000
Recombinant mouse FGFR1 beta (IIIb) Fc chimera protein	R & D Systems	765-FR-050	50 nM (LACE) 125nM (Pulldown)

Recombinant mouse FGFR2 beta (IIIb) Fc chimera protein	R & D Systems	708-MF-050	50 nM (LACE)
Recombinant mouse FGFR2 alpha (IIIb) Fc chimera protein	R & D Systems	663-FR-050	125nM (Pulldown)
Recombinant human FGF1 protein	R & D Systems	232-FA-025	50 nM (LACE)<br>125nM (Pulldown)
Recombinant human FGF2 protein	R & D Systems	233-FB-025	125nM (Pulldown)
Recombinant human FGF7 protein	R & D	251-KG-050	50 nM (LACE)<br>125nM (Pulldown)
Recombinant human FGF10 protein	R &D	345-FG-025	50 nM
Heparinase III (heparitinase 1) *Flavobacterium heparinum*	Amsbio LLC	AMS.HEP-ENZ III-S	0.020 IU/mL
Chondroitinase ABC	Amsbio LLC	AMS.E1028-02	0.040 IU/mL
proteinase K solution	Bioline	BIO-37084	0.5 mg/mL
Xylene substitute	Millipore Sigma	A5597	
Mouse on Mouse blocking reagent	Vector Laboratories	MKB-2213-1	1:12.5

**Primers**		
**Gene**	**Forward primer**	**Reverse primer**
Acta2	GCATGGATGGCATCAATCAC	ACCTATCTGGTCACCTGTATGTA
Agr2	ACCCTTGCGGCTCACACA	CCGAGAGTCCTTTGGGTCCTTT
Agrn	CCCACCCTCCGAGCCTACCACAC	ACAGAGCCAGAGCCAGGAAATCTTTGC
Agt	CCGACTAGATGGACACAAGGT	AGCCTGGAGCAGTGAAGAG
Aqp5	TCTACTTCTACTTGCTTTTCCCCTCCTC	CGATGGTCTTCTTCCGCTCCTCTC
Bhlha15 (Mist1)	TCGCTGACCGCCACCATACTTAC	CTGCTGCTGCTGCTGCTGTTG
Ccnd1	CTTAATGTGATTACCGCTGTATTCC	CCTGACTGCTGTGATGCTATG
Ceacam10	AACTGGAACCGAACTCTGGGAAAG	GGTGGAAGGAGGTGAAGCAAGGC
Cldn10	CGGGAACCAGCGAGAGCG	ATGGAGACTACGAAGGCGACGATT
Cnn1	CGCACAACTACTACAACTC	CCCAAACCGTAACCCTATA
Col4a1	GGTGTTGATGGCTTGCCTGGAGAG	GCCTGGTTGCCCTTTGAGTCCTG
Col4a2	GCAGCCTGGTGTACTCGGTCTTCC	TGGTCGCCTTTGGGTCCTTTGGG
Col9a2	GCAATCAATGGCAAGGATGG	TGAGGCGAGCAGAGGTATAG
Col18a1	CCCACCCTCTCACTTGCTCATACTC	CCACGGCTCGGGCTTGCTG
Ctgf	GCGTCCAGACACCAACCT	ACCAAGGCGAGGCTGATG
Egf	CACTGGTCCTGCTGCTCCTCTTGG	CTGCTGCTCACACTTCCGCTTGG
Erbb3	ATGTGACGGGCTCTGAGGCTGAAC	ACGGGAGTAAGCAGGCTGTGTCG
Etv4	CGCACAGACTTCGCCTACGACTCAG	CATAGCCATAACCCATCACTCCATCACCTG
Etv5	AAGCCCTTCAAAGTGATAGCGGAGAC	GTGTCCACAAACTTCCTCTTTCTGTCAATC
Fgf1	GCACCGTGGATGGGACAAGGGACAGGAG	CACTTCGCCCGCACTTTCCGCACTGAG
Fgf7	CAGCCCCGAGCGACACACCAGAAGTTATG	TCCTGGGTCCCTTTCACTTTGCCTCGTTTG
Fgf9	GATAGCCAAACCACTCTCTC	CATGTCCTTCAACACTGCTT
Fgf10	TCTTCCTCCTCCTCGTCCTTCTCCTCTCCTTCC	CCGCTGACCTTGCCGTTCTTCTCAATCG
Fgfr1b	AGAGCGGGGAGTATGTGTGTAAGGTTTC	TGGTGACAGTGAGCCACGCAGAC
Fgfr2b	TGGCTCTGTTCAATGTGACGGAGATGGATG	AGGCGCTTGCTGTTTGGGCAGGAC
Glce	CATTTCTTGAGAGGGAGTGAGCATTTGTTG	GCTTATGTATGTGACCGTGAAACCTGAAC
Gpc3	GAGACTGCGGTGATGATG	TTCTGATTTCCATGCTGCT
Hs3st1	CCATCCGCCTGCTGCTTATCCTGAG	AGCCGACCGTCCCGCATTAGG
Hs3st2	TCCCTCGGTCCTCTGTGCCCTAC	TTCATCTCAGCCAATGTCGTTTCTAAGTCC
Hs3st3a1	GGTGATGTCTCCTCCCTTCCCTGTC	CGTGCTCCTCGCTAAACCAATTTAATTCC
Hs3st3b1	GCGGGCATTGCTGGAGTTCCTG	GGGTTCTGGGCATCAAGTCTCGGTAC
Hs3st4	ACCCAGATGTCATCCATAGACTTCG	TCACTTATCACCCTCTTCCTGTTCC
Hs3st5	CCTCACTGACTGTTGCTCTT	GGAGGAGGTTCGCATTCT
Hs3st6	GCCATTCAACCGCAAGTTCTACCAG	GTGAGCCAGCAGACAGACATAAATTAAAGG
Hs2st1	GCTCTGCTGTCACCTTCCTGCTG	GCCATCTTCCTTAGTCCTCACAACATCC
Hs6st1	TGAGAGGAATTTGTTTAGATGCCCAGTTTAG	TGACAGAAGCAGCAGCAACCAAC
Hs6st2	CGGCGGTGGTGGATGGCAAG	GGCTTTGTGGAGGATGGAGAGTTGG

Hs6st3	CCTGGCTCTTCTCCCGCTTCTCC	AATTCCTGGTGTGGCTGTGGTTGC
Ndst1	TGGCTGGTTTCTGTTTGGATTCTGTTTCTG	AATGGCTGGTGGACACTGGACTGG
Ndst2	ACAGAAGACAGGCACCACGGCTATTC	TGGCATTGGAAGGAACAGGGAAGAAGTC
Ndst3	AGTCTCTGGGCGGGCTTCGG	TCGTAATGATGTCTTGACCAAGGGATGAG
Ndst4	TCTGGTGCCTGGGTGGTATGC	GGTGTAACTCCTAGAAACTTCTGGACTTC
Hspg2	CAGCCGCAGCAAGGACTTCAT	CGATGCCACTCGCCGTCATTG
Kit	CCTCAGCCTCAGCACATAGC	GAACACTCCAGAATCGTCAACTC
Kitl	TCATCCTTACCTGTTCTTGCTACC	GTTCTTGTCCCTATCATCACTTGC
Klk1b21	TGTTACTACGCCTCAGCAAGCCT	GGGATTTGCCATTTCGTGGGTGTA
Klk1b24	CTCACTGCCTGCTGCTCCTG	GAATCCTCCAACCACACGAGACTG
Krt5	TCCTGTTGAACGCCGCTGAC	CGGAAGGACACACTGGACTGG
Krt14	GCTGCTACATGCTGCTCAGGCTTAGG	CCAGGAAGGACAAGGGTCAAGTAAAGAGAGTG
Krt19	GCCACCTACCTTGCTCGGATTG	GTCTCTGCCAGCGTGCCTTC
Lama3	GCGAGAACTGTCCAGATCGGGTGGCAAAGC	AGCATCCACAGCACAACGCACCAGCTCATC
Lama5	GCCGCCAGCAAGGTCAAGGTGTCCATGAAG	GGCAGTGTACGCAGCAAGGTCGGCAAGG
Lamb1	AGATTCCAGCCAGCAGCCGATGTG	AACCTCACCGTGTAGTTCATTCCCTTCTC
Lamb2	GCCCACACCCTGAGCCTGACG	GCTGCCCATCTTCATCCCGACATC
Lamb3	TCAGTGCTATCCCAGACCAAACAAGACATC	CCGACCACATCATCTACCTGCCCTTC
Lamc2	ATTTCTCTATGTTCCAGCCGTCTCTCCAC	AGTAGTCTTCCAAACTGAGGTCCCAATGC
Lpo	TGACCTTGCTCCAGACTG	CCTTGACCTCTTCCACTGT
mKi67	TTGCCTCCTAATACACCACTGA	CCGTTCCTTGATGATTGTCTTGA
Mmp2	CCTGGTGCTCCACTCTTCTGGTTCTTC	CAGTGCCCTCCTAAGCCAGTCTCTATTAAC
Mmp14	CCCTCCCTCCAGCCTCCCTTCTC	GACCGTCTTCTGCTCAGCCCTCAAG
Mmp15	CAAGTGGTGGATTCTTATAG	GCTGATTATCTACAGAACTAA
Mmp16	TGATGGACCAACAGACCGAGATAAAGAAGG	GGCCAAGATGCAGGGAATGACAATAGC
Mucl1	CCTGGCACTCCTTGTGTTGCTTGG	TGTCTCCGCGTCCTCAGTTCCA
Muc13	AGTGAAGCATCATTGAGTGGACA	GGTAGCAGGTGGCGTCTT
Myc	GGAGTGAGCGGACGGTTGGAAGAG	AGCGGCGGCGAGGGTTGC
Myh11	GTCAGGAGCCACAGTCACCAGCAA	GGCAGGCAGGAAAGGGAAGGGAAT
Mylk	CCATCCTGCGGTGTCTCA	AATGTCCTCCTTGCTGTAACTCA
Ndst1	TGGCTGGTTTCTGTTTGGATTCTGTTTCTG	AATGGCTGGTGGACACTGGACTGG
Ngf	TTGATCGGCGTACAGGCAGAACC	CGGAGGGCTGTGTCAAGGGAATG
Ngfr	TCCACACTCCTTCTCTTACACATA	TTGACGCCCTCATTCAGAAAG
Nrg1	GCTCATCACTCCACGACTGTCACC	CTGCTGTGCCTGCTGTTCTCTACC
Ntf5	CGACGACGACAACTATGA	GGACTCACACCTTACATTAG
Ntrk2	GACACGCACTCCGACTGACT	CCAAGACCAGCAGGCATAAGC
Pdgfa	CGCCTCTGCCTTCCTGTGTTCCT	ATCTCTGTCATGTCTCCATGCTGCCATAGA
Postn	CGAGAAATCATCCAACCAGCAGAG	GCTCCCTTTCTTCGCTAGTCATTC
Prol1	ACC ACA CCA GCA ACA ACC ACA A	TGG CTG TAG AGG TGC TAG GCT TAG
Rps29	GGAGTCACCCACGGAAGTTCGG	GGAAGCACTGGCGGCACATG
Tagln	GGTGAGCCAAGCAGACTT	TTGTGGACTGGAAGGAGAG
Tgfb1	CTCCGCATCCCACCTTTG	CGTCAGCACTAGAAGCCA
Tgfb2	CATCTACAACAGTACCAGGGACTT	AAGCGGACGATTCTGAAGTAGG
Tgfb3	CGGGTCAGGGTGTATTCTC	GCCGAGTCATCATGTCAGA
Thbs1	CACCGCCAAACAACCTCTGACAT	AGTACCGAACAGCTCCTCCACATT
Hs3st3a1 ZFN	CTGGCCTTACTTCTGGACGA	CAAGGGAGAAGAACGGGAG
Hs3st3b1 KO	TCACAGCTCCGAATGAGACATC	CCCAGCGCCTACTGTCTTATC
