# Supplementary material for: Specific 3-O-sulfated heparan sulfate domains regulate salivary gland basement membrane metabolism and epithelial differentiation
Source: Nat Commun. 2024 Aug 31;15:7584. doi: 10.1038/s41467-024-51862-0 (PMC11365954; doi:10.1038/s41467-024-51862-0)
Supplement: Supplementary file 3 — Description Of Additional Supplementary Files [file 41467_2024_51862_MOESM3_ESM.pdf]

Description of Additional supplementary file

Supplementary data 1:

This Excel file contains the differentially expressed genes from RNAsequencing analysis of male and female DKO SMGs compared to WT SMGs.
