# Supplementary material for: Specific 3-O-sulfated heparan sulfate domains regulate salivary gland basement membrane metabolism and epithelial differentiation
Source: Nat Commun. 2024 Aug 31;15:7584. doi: 10.1038/s41467-024-51862-0 (PMC11365954; doi:10.1038/s41467-024-51862-0)
Supplement: Supplementary file 5 — Reporting summary [file 41467_2024_51862_MOESM5_ESM.pdf]

nature portfolio

Corresponding author(s): Vaishali N. Patel

Last updated by author(s): 8/7/2024

Reporting Summary

Nature Portfolio wishes to improve the reproducibility of the work that we publish. This form provides structure for consistency and transparency in reporting. For further information on Nature Portfolio policies, see our Editorial Policies and the Editorial Policy Checklist.

Statistics

For all statistical analyses, confirm that the following items are present in the figure legend, table legend, main text, or Methods section.

n/a	Confirmed	
☐	☒	The exact sample size (*n*) for each experimental group/condition, given as a discrete number and unit of measurement
☐	☒	A statement on whether measurements were taken from distinct samples or whether the same sample was measured repeatedly
☐	☒	The statistical test(s) used AND whether they are one- or two-sided<br>*Only common tests should be described solely by name; describe more complex techniques in the Methods section.*
☒	☐	A description of all covariates tested
☐	☒	A description of any assumptions or corrections, such as tests of normality and adjustment for multiple comparisons
☐	☒	A full description of the statistical parameters including central tendency (e.g. means) or other basic estimates (e.g. regression coefficient) AND variation (e.g. standard deviation) or associated estimates of uncertainty (e.g. confidence intervals)
☐	☒	For null hypothesis testing, the test statistic (e.g. *F*, *t*, *r*) with confidence intervals, effect sizes, degrees of freedom and *P* value noted<br>*Give P values as exact values whenever suitable.*
☒	☐	For Bayesian analysis, information on the choice of priors and Markov chain Monte Carlo settings
☒	☐	For hierarchical and complex designs, identification of the appropriate level for tests and full reporting of outcomes
☒	☐	Estimates of effect sizes (e.g. Cohen's *d*, Pearson's *r*), indicating how they were calculated

Our web collection on statistics for biologists contains articles on many of the points above.

Software and code

Policy information about availability of computer code

Data collection	qPCR results were collected using a C1000 Touch Thermal Cycler and CFX96 Real-Time System connected with the Bio-Rad CFX Maestro 2.3 Software (All from Bio-Rad).<br>All confocal images of IHC stains were captured using a Zeiss LSM 880 Confocal Laser Scanning Microscope (40X and 63X objectives) and ZEN Miscroscopy Software or Nikon A1R+ MP microscope (20x objective) and the Confocal NIS-Elements Package (Nikon instruments).<br>Slides with H&E stained slides were scanned using a S60 NanoZoomer Digital (Hamamatsu).<br>Wester blots were imaged on a GE Amersham Imager AI680 using automatic exposure settings.
Data analysis	IHC and Western Blot quantification was done using FIJI and further analysis and calculations were done using Microsoft Excel and Prims 9.<br>Images from the S60 Nanozoomer Digital slide scanner was exported using the NDP.view 2 software (Hamamatsu).<br>Analysis of qPCR results were done using Microsoft excel and Prism10.<br>All scRNAseq data was analyzed using R and R studio (https://rstudio.com/) and Seurat V4. No custom functions or code was used for the analysis in this work. For RNAseq data, differential gene expression was analyzed using DESeq2.

For manuscripts utilizing custom algorithms or software that are central to the research but not yet described in published literature, software must be made available to editors and reviewers. We strongly encourage code deposition in a community repository (e.g. GitHub). See the Nature Portfolio guidelines for submitting code & software for further information.

Data

Policy information about availability of data

All manuscripts must include a data availability statement. This statement should provide the following information, where applicable:

- Accession codes, unique identifiers, or web links for publicly available datasets
- A description of any restrictions on data availability
- For clinical datasets or third party data, please ensure that the statement adheres to our policy

All scRNAseq used in this study are from previously deposited data. Murine SMG at E16 developmental stages is from GSE150327 [https://www.ncbi.nlm.nih.gov/geo/query/acc.cgi?acc=GSE150327]. Ready to use .rds objects from publicly available scRNAseq data of embryonic SMGs were downloaded (GSE150327, https://sgmap.nidcr.nih.gov). RNAseq data for male and female SMGs from WT and Hs3st3a1; Hs3st3b1 double knockout mice is avaiable on the Gene Expression Omnibus (GEO) website (GEO: GSE235187).
Specific values used to generate graphs in this paper are provided in the Source data file.

Field-specific reporting

Please select the one below that is the best fit for your research. If you are not sure, read the appropriate sections before making your selection.

☒ Life sciences ☐ Behavioural & social sciences ☐ Ecological, evolutionary & environmental sciences

For a reference copy of the document with all sections, see nature.com/documents/nr-reporting-summary-flat.pdf

Life sciences study design

All studies must disclose on these points even when the disclosure is negative.

Sample size	Sample size for each experiment was determined based on our previous work and the number of specific genotypes we could logistically generate. We included a minimum of 3 biological replicates for each group/treatment/timepoint/sex.
Data exclusions	No data was excluded from analysis.
Replication	Organ and epithelial culture experiments were replicated using embryos from at least 3 independent litters.<br>IHC staining and qPCR was repeated on at least 3 biological replicates fro each genotype/sex.
Randomization	Experiments with embryonic or neonatal glands, embryos were genotyped and data was collected.<br>Experiments using adult mice with specific genotypes, animals were used based on genotyping results and at least 3 animals were used per group/sex. Experiments using adult mice with specific genotypes, animals were used based on genotyping results and at least 3 animals were used per group/sex.<br>For organ culture experiments using isolated epithelia, four epithelia from a pool of isolated epithelia from separate embryos were placed in laminin-111 matrix on a filter/dish and randomly treated with 12 mer HS. These experiments were repeated at least three times using independent litters.<br>Myoepithelial cultures were performed on at least 5 independent litters of 4-6 pups from WT or homozygous DKO crosses on postnatal mice. Three-four replicates were set up for each experiment.
Blinding	Samples used for IHC were picked based on genotyping results and all quantification of histological images were performed blinded.<br>Heparan sulfate analysis of submandibular gland samples was performed on blinded samples.

Reporting for specific materials, systems and methods

We require information from authors about some types of materials, experimental systems and methods used in many studies. Here, indicate whether each material, system or method listed is relevant to your study. If you are not sure if a list item applies to your research, read the appropriate section before selecting a response.

Materials & experimental systems

n/a	Involved in the study	
☐	☒	Antibodies
☒	☐	Eukaryotic cell lines
☒	☐	Palaeontology and archaeology
☐	☒	Animals and other organisms
☒	☐	Human research participants
☒	☐	Clinical data
☒	☐	Dual use research of concern

Methods

n/a	Involved in the study	
☒	☐	ChIP-seq
☒	☐	Flow cytometry
☒	☐	MRI-based neuroimaging

Antibodies

Antibodies used

Heparan sulfate proteoglycan (perlecan) (1:200, Millipore Sigma, mAb1948, E-cadherin (1:100, Cell signaling, mAb#3195, E-cadherin (1:200, BD Biosciences, #610182, Smooth muscle actin (SMA) (1:200, Millipore Sigma, A2547), Aquaporin 5 (AQP5) (1:100, Alomone Labs, AQP-005), Acinar-1 (1:100, DSHB, 3.7A12), Calponin (CNN1) (1:100, Abcam, ab46794), Keratin 14 (1:2000, Covance, PRB-155P), Mucin 13 (1:100, Santa Cruz Biotechnology, Sc-390115), Mucin 10 /Prol1 (1:200, Everest Biotech, EB10617), Collagen TIV (1:200, Millipore, AB769), Collagen TIV (1:200, EMD Millipore, AB756), NGF (1:100, Alomone Labs, AN-240), Agrin (1:100, R &D, AF550), p44/p42 (Erk1/2) (1:1000, Cell Signaling, #9102), phospho-p44/p42 MAPK (Erk1/2) (1:100 (IF), 1:1000 (WB), Cell Signaling, #4370), tight junction protein 1 ZO-1 (1:200, Invitrogen, #339100), Delta heparan sulfate 3G10 (1:200 (IF), 1:2000 (WB), Asmbio, 370260-1), EGF (1:100, Dr. Edward W. Gresik), beta-actin (1:5000, Santa Cruz, Sc-477780, HS4C3V single-chain HS Ab (1:50, Dr. Toin H. van Kuppevelt (Radboud University Medical Center, The Netherlands), Anti-VSV glycoprotein-Cy3 (1:100, Sigma, C7706), HRP-linked secondary antibodies for western blotting (1: 10,000, Cell Signaling) and Beta-actin (13E5) Rabbit mAb (HRP conjugate) (1:1000, Cell Signaling, #5125S). Secondary antibodies used for immunostaining were Alexa Fluor® 488 AffiniPure F(ab')$_2$ Fragment Donkey Anti-Goat IgG (H+L) (1:200, #705-546-147), Alexa Fluor® 647 AffiniPure F(ab')$_2$ Fragment Donkey Anti-Goat IgG (H+L) (1:200, #705-606-147), Cy™3 AffiniPure F(ab')$_2$ Fragment Donkey Anti-Rabbit IgG (H+L) (1:200, #711-166-152) all from Jackson Immunoresearch Laboratories.

Validation

All but two antibodies used in this study were purchased from vendors and validation for their applicability can be found at the vendors websites. The antibodies have also been confirmed by others and used in multiple publications. We also optimized protocols and dilutions and performed secondary only controls for these antibodies.
Anti-heparan sulfate proteoglycan (perlecan) (Millipore Sigma, mAb1948) has been tested by the company and used in several publications including Patel et.al., 2007.
Anti-E-cadherin (Cell Signaling, mAb#3195) has been validated by the vendor and used in several publications including Chibly et al., 2023.
Anti-calponin (Abcam, ab46794) has been validated by the vendor and has been used in Chibly et al., 2023.
Anti-smooth muscle actin (Millipore Sigma, A2547) has been validated by the vendor and used in several publications including Patel et al., 2021.
E-cadherin (BD Biosciences, #610182) has been tested by the company and used in several publications including Aure et al., 2023.
Aquaporin 5 (Alomone Labs, AQP-005) has been validated by the vendor and used in peer-reviewed publications including Aure et al., 2023.
Muc10/Prol1 (Everest, EB10617) has been validated by the vendor and used in several peer-reviewed publications including Aure et al., 2023 and Peluso et al., 2019.
ZO-1 (Invitrogen, #339100) has been validated by the vendor and has been used in several publications including Grosse et al., 2011.
Phospho-p44/42 MAPK (Cell signaling, #4370) has been validated by the vendor and used in several publications including Aure et al., 2023 (Nature Communications) and Seidel et al., 2016 (Oncotarget).
p44/p42 (Erk1/2) (Cell signaling, #9102) has been validated by the vendor and used in several publications including Aure et al., 2023.
Acinar-1 antibody (DBHB, #3.7A12) has previously been used in a number of peer-reviewed publications including Peluso et al., 2019.
Keratin 14 (Covance, PRB-155P) has been validated by the vendor and used in several peer-reviewed publications including Patel et al., 2014.
NGF (Alomone Labs, #AN-240) has been validated by the vendor and has been used in several publications including Chibly et al., 2023.
Collagen TIV (Millipore Sigma, #AB769) has been validated by the vendor and has been used in several publications including Patel et al., 2021.
Agrin (R &D, AF550) has been validated by the vendor and has been used in peer-reviewed publications including Dora et al., 2021 (Cellular and Molecular Gastroenterology and Hepatology).
Beta-actin (Santa cruz, #sc-47778) has been validated by the vendor and has been used in several peer-reviewed publications including Li et al., 2023 (iScience).
Delta-heparan sulfate 3G10 (Asmbio LLC, #370260-1) has been validated by the vendor and has been used in several peer-reviewed publications including Montoliu-Gaya et al., 2021 (Western Blots) and Wei et al., 2019 (Immunostaining). Anti-VSV glycoprotein-Cy3 has been used in Patel et al., 2021.
B-actin (13E5) Rabbit mAb (HRP Conjugate) #5125 has been validated by the vendor and has been used in several publications including Bloch et al., 2021 (Nature Communications).
EGF antibody was kindly provided by Dr. Edward W. Gresik and has previously been used in a peer-reviewed publication Mauduit and Aure et al., 2022 (Cell Reports).
HS4C3V single-chain HS Ab has been used in several publications including Patel et al., 2021.

Animals and other organisms

Policy information about studies involving animals; ARRIVE guidelines recommended for reporting animal research

Laboratory animals

Strains used:
Hs3st1 KO (Thackerat al., 2016, Shrowak et al., 2002).
Hs3st3a1 KO (Patel et al., 2021)
Hs3st3b1 KO (Patel et al., 2021)
Hs3st3a1; Hs3st3b1 DKO (Wang et al., 2023)
Timed pregnant ICR (CD-1) females (Envigo)
Due to the known sexual dimorphism in adult mouse SMGs, differing outcomes between sexes was considered when using adult mice and findings in this work apply to both sexes. Sex was not considered in experiments using embryonic or neonatal glands since no known sexual dimorphism is present during development.
Adult mice used were 2-4 months old.
Glands at various developmental stages were E13, E15, E16 or postnatal as indicated for specific experiments.
Mice were kept in a 14hr on/10hr off light/dark cycle at 74-78 F with 50-70% humidity.

Wild animals	This study did not use wild animals.
Field-collected samples	No field-collected samples.
Ethics oversight	All experiments were approved by the NIH Animal Care and Use Committee.

Note that full information on the approval of the study protocol must also be provided in the manuscript.
